# Notoginsenoside R1 promotes Lgr5^+^ stem cell and epithelium renovation in colitis mice via activating Wnt/β-Catenin signaling

**DOI:** 10.1038/s41401-024-01250-7

**Published:** 2024-03-15

**Authors:** Zhi-lun Yu, Rui-yang Gao, Cheng Lv, Xiao-long Geng, Yi-jing Ren, Jing Zhang, Jun-yu Ren, Hao Wang, Fang-bin Ai, Zi-yi Wang, Bei-bei Zhang, Dong-hui Liu, Bei Yue, Zheng-tao Wang, Wei Dou

**Affiliations:** 1 The MOE Key Laboratory of Standardization of Chinese Medicines, Shanghai Key Laboratory of Compound Chinese Medicines, and the SATCM Key Laboratory of New Resources and Quality Evaluation of Chinese Medicines, Institute of Chinese Materia Medica, Shanghai University of Traditional Chinese Medicine (SHUTCM), Shanghai, 201203 China; 2 Centre for Chinese Herbal Medicine Drug Development Limited, Hong Kong Baptist University, Hong Kong SAR, China

**Keywords:** inflammatory bowel disease, notoginsenoside R1, intestinal stem cells, Wnt/β-Catenin pathway, ICG-001

## Abstract

Inflammatory bowel disease (IBD) is characterized by persistent damage to the intestinal barrier and excessive inflammation, leading to increased intestinal permeability. Current treatments of IBD primarily address inflammation, neglecting epithelial repair. Our previous study has reported the therapeutic potential of notoginsenoside R1 (NGR1), a characteristic saponin from the root of *Panax notoginseng*, in alleviating acute colitis by reducing mucosal inflammation. In this study we investigated the reparative effects of NGR1 on mucosal barrier damage after the acute injury stage of DSS exposure. DSS-induced colitis mice were orally treated with NGR1 (25, 50, 125 mg·kg^−1^·d^−1^) for 10 days. Body weight and rectal bleeding were daily monitored throughout the experiment, then mice were euthanized, and the colon was collected for analysis. We showed that NGR1 administration dose-dependently ameliorated mucosal inflammation and enhanced epithelial repair evidenced by increased tight junction proteins, mucus production and reduced permeability in colitis mice. We then performed transcriptomic analysis on rectal tissue using RNA-sequencing, and found NGR1 administration stimulated the proliferation of intestinal crypt cells and facilitated the repair of epithelial injury; NGR1 upregulated ISC marker Lgr5, the genes for differentiation of intestinal stem cells (ISCs), as well as BrdU incorporation in crypts of colitis mice. In NCM460 human intestinal epithelial cells in vitro, treatment with NGR1 (100 μM) promoted wound healing and reduced cell apoptosis. NGR1 (100 μM) also increased Lgr5^+^ cells and budding rates in a 3D intestinal organoid model. We demonstrated that NGR1 promoted ISC proliferation and differentiation through activation of the Wnt signaling pathway. Co-treatment with Wnt inhibitor ICG-001 partially counteracted the effects of NGR1 on crypt Lgr5^+^ ISCs, organoid budding rates, and overall mice colitis improvement. These results suggest that NGR1 alleviates DSS-induced colitis in mice by promoting the regeneration of Lgr5^+^ stem cells and intestinal reconstruction, at least partially via activation of the Wnt/β-Catenin signaling pathway.

**Figure:**
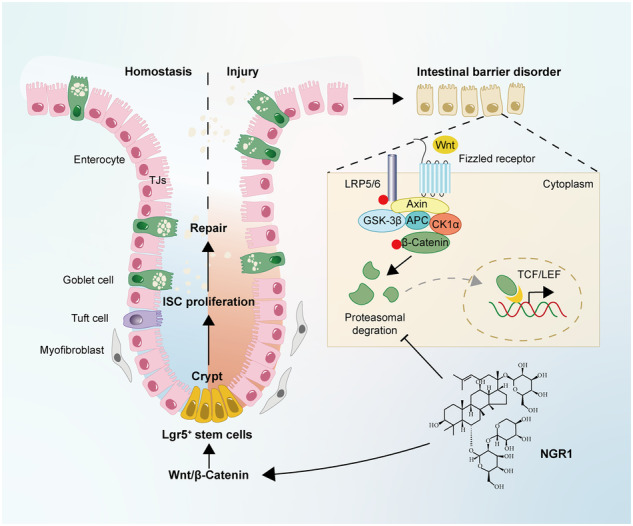
Schematic diagram of the mechanism of NGR1 in alleviating colitis. DSS caused widespread mucosal inflammation epithelial injury. This was manifested by the decreased expression of tight junction proteins, reduced mucus production in goblet cells, and increased intestinal permeability in colitis mice. Additionally, Lgr5^+^ ISCs were in obviously deficiency in colitis mice, with aberrant down-regulation of the Wnt/β-Catenin signaling. However, NGR1 amplified the expression of the ISC marker Lgr5, elevated the expression of genes associated with ISC differentiation, enhanced the incorporation of BrdU in the crypt and promoted epithelial restoration to alleviate DSS-induced colitis in mice, at least partially, by activating the Wnt/β-Catenin signaling pathway.

Schematic diagram of the mechanism of NGR1 in alleviating colitis. DSS caused widespread mucosal inflammation epithelial injury. This was manifested by the decreased expression of tight junction proteins, reduced mucus production in goblet cells, and increased intestinal permeability in colitis mice. Additionally, Lgr5^+^ ISCs were in obviously deficiency in colitis mice, with aberrant down-regulation of the Wnt/β-Catenin signaling. However, NGR1 amplified the expression of the ISC marker Lgr5, elevated the expression of genes associated with ISC differentiation, enhanced the incorporation of BrdU in the crypt and promoted epithelial restoration to alleviate DSS-induced colitis in mice, at least partially, by activating the Wnt/β-Catenin signaling pathway.

## Introduction

Inflammatory bowel disease (IBD), which includes Crohn’s disease (CD) and ulcerative colitis (UC), represents two major idiopathic gastrointestinal conditions characterized by chronic and recurrent inflammation, primarily leading to progressive mucosal damage [1]. The incidence and prevalence of IBD are on the rise globally, particularly in regions like South America and Asia [2]. The exact etiology of IBD remains complex and elusive, with major contributing factors believed to involve disruptions in the mucosal barrier, alterations in the gut microbiota, and prolonged immune responses influenced by both genetic predisposition and environmental factors [3, 4].

A predominant hallmark of IBD is the persistent injury to the mucosal barrier. This injury is characterized by increased intestinal permeability, damage to epithelial cells, and mucosal inflammation [5]. This barrier dysfunction allows luminal antigens and microbial products to breach the intestinal epithelium, triggering immune cells in the lamina propria to release pro-inflammatory cytokines and chemokines, further exacerbating mucosal damage [6]. Therefore, an effective approach to treating IBD should not only focus on managing inflammation but should also prioritize the restoration of the intestinal mucosal barrier function [5]. However, existing treatments for IBD, such as mesalamine, immunosuppressants, and anti-cytokine biological agents, primarily target inflammation in the gut mucosa of IBD patients, with less emphasis on repairing mucosal barrier damage. This has resulted in limited success in healing intestinal ulcers [1].

The overall health of the mucosal barrier relies on the integrity of the epithelium, which is maintained through continuous regeneration, renewal, and differentiation by intestinal stem cells (ISCs) located in the crypts of the intestine [7]. These ISCs play a crucial role in proliferating and differentiating into various cell types, including absorptive- and secretory-lineage cells, to ensure the integrity of the intestinal mucosa [8]. Lgr5 is a widely recognized marker of ISCs, responsible for generating clonal cell populations and forming the entire colonic crypt [9]. However, disruptions in the process of ISC proliferation and differentiation can impede the coordinated restoration of damaged intestinal tissues, leading to significant barrier defects [10]. Moreover, individuals with UC may exhibit a shortage of goblet cells derived from ISCs, further compromising the physical barrier that normally guards against microbial contact [11]. Recent preclinical studies have emphasized the urgent need for “mucosal barrier restitution” to achieve long-term remission in IBD patients [12]. This approach holds great promise for effectively treating patients with UC and CD by rectifying the aberrant proliferation and differentiation of ISCs while reducing excessive inflammation.

The Wingless/Int1 (Wnt) pathway is a well-documented regulator of ISCs proliferation [13]. This pathway has been extensively studied, with numerous comprehensive reviews exploring how the Wnt/β-Catenin signaling pathway governs ISCs proliferation, differentiation, and the maintenance of intestinal barrier function and repair [14]. Research has also suggested that dysregulation of Wnt signaling leads to a deficiency of intestinal secretory cells, resulting in a compromised mucus barrier and, consequently, the breakdown of the intestinal barrier in UC patients [15]. Notably, the aberrant suppression of the Wnt signaling pathway can induce abnormal gene expression and epigenetic modifications, both of which are essential factors contributing to the genetic susceptibility to IBD [16, 17]. Additionally, studies have revealed that the crosstalk between the Wnt and inflammatory signaling pathways mediates the production of inflammatory cytokines, such as IL-6 and IL-8, during the pathogenesis of colitis [18]. As a result, the Wnt/β-Catenin signaling pathway has emerged as a promising target for drug development and personalized medicine, holding great potential for improving patient outcomes.

Notoginsenoside R1 (NGR1), a distinctive saponin isolated from the root of *Panax notoginseng* [19], is a novel phytoestrogen with a wide range of potential therapeutic applications, including in cardiovascular, neurological, and metabolic diseases [20–22]. Furthermore, NGR1 has demonstrated protective effects on various organs, such as the liver, kidney, and lungs, suggesting its potential utility in treating organ damage [23]. At the molecular level, NGR1 has been shown to stimulate proliferation and enhance tube formation in human umbilical vein endothelial cells by activating the Wnt/β-Catenin signaling pathway [24]. Our previous study has revealed that NGR1 alleviates acute colitis in mice by inhibition of mucosal inflammation [25]. In this study, we assessed the reparative effects of NGR1 on mucosal barrier damage after the acute injury stage of DSS exposure. We hypothesize that the mechanism underlying these effects, at least in part, is associated with the activation of the Wnt/β-Catenin signaling pathway.

## Materials and methods

### Chemicals and reagents

Notoginsenoside R1 (NGR1, BP1010, C_47_H_80_O_18_, purity ≥98%, CAS No 80418-24-2, MW: 933.13 Da) was purchased from Chengdu Purifa Technology Development Co. Ltd (Chengdu, China). Dextran sulfate sodium salt (DSS, 0216011010, MW: 36 kDa–50 kDa) was purchased from MP Biomedicals (Shanghai, China). Salicylazosulfapyridine (SASP, S0883, C_18_H_14_N_4_O_5_S, CAS No 599-79-1, MW: 398.39 Da) and FITC-dextran (FD4, CAS No 60842-46-8) was purchased from Sigma-Aldrich (Darmstadt, Germany). ICG-001 (T6113, C_33_H_32_N_4_O_4_, purity ≥ 98%, CAS No 780757-88-2, MW: 548.64 Da) was acquired from TOPSCIENCE (Shanghai, China). Water-DEPC treated (693520) and DMSO (D8418) were obtained from MilliporeSigma (Burlington, MA, USA).

### Cell culture

NCM460 human intestinal epithelial cells and CT26 murine colon carcinoma cells were obtained from the American Type Culture Collection (ATCC, Manassas, VA, USA). NCM460 and CT26 cells were cultured in Roswell Park Memorial Institute (RPMI)-1640 culture medium (11875085, Gibco, NY, USA) supplemented with 10% fetal bovine serum (10099158, Gibco, NY, USA). The culture conditions included a humidified atmosphere containing 5% CO_2_, with a constant temperature maintained at 37 °C.

### Animals

The Laboratory Animal Center of Shanghai University of Traditional Chinese Medicine provided female C57BL/6 mice weighing 20 ± 2 g. These mice were housed in a specific pathogen-free facility under meticulously controlled conditions, including a temperature range of 23–25 °C, humidity maintained at 60%–70%, and a well-regulated 12-h light-dark cycle. The Animal Experimentation Ethics Committee of Shanghai University of Traditional Chinese Medicine granted approval (PZSHUTCM2307310004) for experimental procedures conducted on the animals. All experiments were conducted in accordance with institutional animal care guidelines and protocols approved by the committee.

### Establishment of acute colitis mouse model

According to the method reported by Yue [26], we established the acute colitis mouse model. Briefly, female C57BL/6 mice were divided randomly into four groups: Control, DSS, DSS + SASP, and DSS + NGR1. Acute colitis was induced by administering 3% DSS in the drinking water of mice for a period of 8 days. Mice in the DSS + SASP group were treated orally with SASP (260 mg/kg) once per day for the same duration. The DSS + NGR1 group received NGR1 (25, 50, 125 mg/kg) by oral gavage once per day for 10 days. Mice in the Control and DSS groups were administered the same volume of Control. Daily monitoring of body weight and rectal bleeding was conducted throughout the 10-day period. At the end of the experiment, mice were euthanized, and the colon was collected for further analysis.

Female C57BL/6 mice were randomly divided into four groups: DSS, DSS + ICG-001, DSS + NGR1 and DSS + ICG-001 + NGR1. To establish an acute enteritis model, mice were subjected to the protocol described above. Mice in the DSS + NGR1 and DSS + ICG-001 + NGR1 group were given NGR1 (25 mg/kg) orally once daily for 10 consecutive days. Meanwhile, mice in the DSS + ICG-001 and DSS + ICG-001 + NGR1 groups were given ICG-001 (20 mg/kg) via intraperitoneal injection three times per week. The DSS and DSS + NGR1 groups received the same volume of Control.

### Xenograft tumor transplantation model

Male BALB/c mice were acclimated for 1 week in a specific pathogen-free environment. Subsequently, CT26 cells (2 × 10^5^ cells/mouse) were subcutaneously transplanted into the left axillary region of each mouse. Once the tumor size reached 200 mm^3^, the mice were randomly assigned to the vehicle group or the NGR1 group based on tumor size. Throughout the 18-day experiment, mice in the vehicle group received 0.5% CMC-Na, while those in the NGR1 group were administered 25 mg/kg NGR1. Tumor volume = 0.5 × length (mm) × width (mm)^2^.

### Measurement of intestinal permeability

C57BL/6 mice were fasted for 4 h before execution. Mice were then orally administered 60 mg/100 g body weight of FITC-dextran in 200 µL of sterile saline. After 4 h, blood samples were collected via retro-orbital bleeding, and serum was separated by centrifugation. The serum FITC-dextran levels were measured at an excitation wavelength of 485 nm and an emission wavelength of 528 nm using a fluorometer (VARIOSKAN FLASH, Thermo Fisher, MA, USA).

### H&E staining

Colonic tissues were collected from mice and fixed in 4% paraformaldehyde. Tissues were then dehydrated, embedded in paraffin, and sectioned into 4 μm thick slices. The sections were then stained with hematoxylin and eosin (H&E) using standard protocols. Stained sections were analyzed under a light microscope (BX61VS, Olympus, Tokyo, Japan), and images were captured for further analysis.

### Enzyme-linked immunosorbent assay (ELISA)

The concentrations of DAO (CSB-E10090m) and LPS (CSB-E13066m) in mouse serum samples were determined using the respective ELISA kit (Wuhan Huamei Biological Engineering Co., Ltd, Wuhan, China). Specifically, serum samples were added to a 96-well plate coated with DAO or LPS-specific antibodies, followed by incubation with detection reagents and substrate solution. Absorbance was measured at 450 nm, and concentrations were calculated using standard curves.

### Immunofluorescent staining

Colonic tissues were fixed in 4% paraformaldehyde, embedded in OCT compound, and sectioned into 5-μm slices. After permeabilization and blocking, sections were incubated with primary antibodies against ZO-1 (#13663, Cell Signaling Technology, CST, MA, USA) and Occludin (#91131, CST, MA, USA), followed by secondary antibodies conjugated to fluorophores (9300039001, ABclonal, Wuhan, China). Nuclei were counterstained with DAPI (#4083, CST, MA, USA), and images were obtained using a fluorescence microscope (BX61VS, Olympus, Tokyo, Japan). Quantification of ZO-1 and Occludin expression was performed using ImageJ software (NIH, Bethesda, MD, USA).

### Alcian blue staining

Colonic tissue samples were obtained from mice, fixed, dehydrated, embedded in paraffin blocks, sectioned, and stained with Alcian blue using a commercial kit. Under a light microscope (BX61VS, Olympus, Tokyo, Japan), the stained sections were examined and images were captured for subsequent analysis.

### Real-time fluorescence quantitative polymerase chain reaction (RT-qPCR)

RNA was extracted using the TRIzol method, and RNA quantity and purity were measured by NanoDrop spectrophotometer (Thermo Fisher Scientific). The RNA was then reverse-transcribed using an Evo M-MLV RT Premix for qPCR kit (AG11706, Accurate Biotechnology Co., Ltd., Chengdu, China), and qPCR was performed using a SYBR^*®*^ Green Premix × *Pro Taq* HS qPCR Kit (AG11718, Accurate Biotechnology Co., Ltd., Chengdu, China) (Table 1). The amplification was carried out using an ABI Prism 7900HT Sequence Detection System (Life Technologies, CA, USA), and data were analyzed using the 2^−ΔΔCt^ method.

**Table 1:** Genes and associated primer sequences used for RT-qPCR analysis.

Primers	Forward primer (5′-3′)	Reverse primer (5′-3′)
m Muc2	AGGGCTCGGAACTCCAGAAA	CCAGGGAATCGGTAGACATCG
m ZO-1	GCCGCTAAGAGCACAGCAA	TCCCCACTCTGAAAATGAGGA
m Occludin	ATGTCC GGCCGATGCTCTC	TTTGCTGCTCTTGGGTCTGTAT
m GAPDH	GGCCGAGAATGGGAAGCTTGT	ACATACTCAGCACCGGCCTCA
m c-Myc	ATGCCCCTCAACGTGAACTTC	CGCAACATAGGATGGAGAGCA
m Cyclin D	GCGTACCCTGACACCAATCTC	CTCCTCTTCGCACTTCTGCTC
m c-Jun	CCTTCTACGACGATGCCCTC	GGTTCAAGGTCATGCTCTGTTT
m Cdx4	TGACATGACCTCCCCAGTTTT	GCCGGAGTCAAGAGAAACCA
m Lgr5	CCTACTCGAAGACTTACCCAGT	GCATTGGGGTGAATGATAGCA
m Olfm4	CAGCCACTTTCCAATTTCACTG	GCTGGACATACTCCTTCACCTTA
m Bmi1	ATCCCCACTTAATGTGTGTCCT	CTTGCTGGTCTCCAAGTAACG
m Ascl2	AAGCACACCTTGACTGGTACG	AAGTGGACGTTTGCACCTTCA
m Sox9	GAGCCGGATCTGAAGAGGGA	GCTTGACGTGTGGCTTGTTC
m Tert	GCACTTTGGTTGCCCAATG	GCACGTTTCTCTCGTTGCG
m Hopx	AGACGCAGAAATGGTTTAAGC	TCCAAGAGCAAGCTCAAGGG
m Lyz1	GAGACCGAAGCACCGACTATG	CGGTTTTGACATTGTGTTCGC
m Gip	TGAGTTCCGATCCCATGCTAA	CCAGTTCACGAAGTCTTGTTGTC
m Sglt1	ATGCGGCTGACATCTCAGTC	ACCAAGGCGTTCCATTCAAAG
m Vill	ACCTTTACCGGGTGGTTTGTC	GGAGTTAGCTGGAAGTTATGGAC
m Sis	GCAAATGGTGCCGAATATCAAAC	TCTGGGTACACTGTTAATCCTGG
m GSK-3β	TGGCAGCAAGGTAACCACAG	CGGTTCTTAAATCGCTTGTCCTG
m β-Catenin	ATGGAGCCGGACAGAAAAGC	CTTGCCACTCAGGGAAGGA

### Western blot

Colonic tissues were extracted and homogenized, and protein was obtained using RIPA lysis buffer with phosphatase and protease inhibitors. Protein concentration was measured using a BCA assay kit (20201ES76, Yeasen Biotech Co., Ltd, Shanghai, China). Equal amounts of protein were loaded onto SDS-PAGE (sodium dodecyl sulfate polyacrylamide gel electrophoresis) gels and separated by electrophoresis. Subsequently, the separated proteins were transferred onto PVDF membranes (000025736, Milipore, MA, USA). The membrane was then blocked with 5% BSA solution for 2 h. After blocking, the membrane was incubated with primary antibodies overnight at 4 °C, followed by incubation with HRP-conjugated secondary antibodies for 1 h at room temperature. Protein bands were visualized using ECL reagents (WBKLS0500, Millipore) and imaged with a GS-700 imaging densitometer (Bio-Rad, CA, USA). Protein expression levels were quantified using ImageJ software (NIH, Bethesda, MD, USA). The following primary antibodies were used: rabbit anti-β-Catenin (1:1000, #8480, CST, MA, USA), rabbit anti-p-GSK-3β (1:1000, #5558, CST, MA, USA), rabbit anti-GSK-3β (1:1000, #12456, CST, MA, USA), rabbit anti-Cyclin D1 (1:1000, #2922, CST, MA, USA), rabbit anti-c-Myc (1:1000, #5605, CST, MA, USA) and rabbit anti-β-actin (1:1000, #4970, CST, MA, USA).

### Immunohistochemistry (IHC)

Colonic tissue sections were fixed in 4% paraformaldehyde, embedded in paraffin, and sliced into 5 μm thick sections. Antigen retrieval was performed using citrate buffer solution (pH = 6.0) and heating in a microwave oven. Non-specific binding was blocked with 5% goat serum for 30 min. Sections were incubated overnight at 4 °C with primary antibodies, followed by incubation with a secondary antibody and staining with DAB (3,3′-diaminobenzidine). Hematoxylin was used for counterstaining before the sections were examined microscopically and images were captured.

### RNA-sequencing

Total RNA was extracted from mouse intestinal tissues using TRIzol reagent according to the manufacturer’s instructions. The extracted RNA was evaluated for quality using a NanoDrop spectrophotometer (Thermo Fisher). RNA sequencing libraries were then constructed with the NEBNext Ultra RNA Library Prep Kit for Illumina, and sequencing was performed on an Illumina HiSeq platform. The differential gene was carried out on the cloud platform of majorbio (https://www.majorbio.com/).

### Transepithelial electrical resistance (TEER)

Caco-2 cells were seeded in Millicell inserts of 24-well plates at a density of 5 × 10^4^ cells/400 μL per well. The outer chamber was filled with 600 μL DMEM medium (2323012, Gibco, NY, USA) and replaced every other day. TEER values were measured using a MERS00002 volt-ohm meter system (Milipore), and the electrode was sterilized with 70% ethanol and rinsed with sterile phosphate-buffered saline (PBS) before each measurement. Monolayer formation was assumed at TEER values of 400 Ω/cm^2^. Measurements were taken at regular intervals using the same electrode and recorded.

### Organoid extraction, culture and treatment

The intestinal crypts were isolated from the small intestine of C57BL/6 mice (6- to 8-week-old). The small intestine was removed and flushed with ice-cold PBS. The intestine was opened longitudinally and cut into 2- to 3-mm pieces. The pieces were then washed with ice-cold PBS and incubated in 3 mM EDTA solution at 4 °C for 20 min with gentle shaking. After incubation, the crypts were released by vigorously shaking the tubes. The supernatant containing the crypts was collected and filtered through a 70-μm cell strainer. The crypts were then centrifuged at 1200 r/min for 5 min and resuspended in Matrigel (Corning, NA, USA). The crypt-Matrigel mixture was plated in 24-well plates and incubated at 37 °C for 30 min to allow the Matrigel to solidify. The IntestCult^TM^ OGM Mouse Basal Medium (#06005, STEMCELL, Vancouver, Canada) was then added to the wells and changed every other day.

After cultured 2 days in a 24 well plate, the intestinal crypts were randomly divided into control, DSS model group and DSS + NGR1 group. Then, the organoids were administered DSS (20 μg/mL), DSS (20 μg/mL) plus NGR1 (100 μM) for 4 days. The organoid growth conditions were recorded by the microscope (Olympus CKX4, Tokyo, Japan). IHC assay was conducted to examine the fluorescent protein expression of Lgr5 and β-Catenin (refer to the above method of IHC).

### Molecular docking

The molecular docking was performed using AutoDock Vina software. The 3D crystal structure of β-Catenin protein (PDB: 1JDH) was obtained from the Protein Data Bank (PDB) database. The structure of NGR1 was drawn and optimized using ChemDraw software and converted to a PDB file using Open Babel software. The protein and ligand files were prepared using AutoDock Tools. Docking simulations were performed and the conformation with the lowest binding energy was selected as the final docking result. The docking results were analyzed using PyMOL software.

### TOPFlash

The TOPFlash assay was performed as previously described with slight modifications [27]. HEK293T cells were seeded in 24-well plates and cultured overnight. The cells were transfected with the 500 ng TOPFlash luciferase reporter plasmids (Beyotime Biotechnology, Shanghai, China) and 50 ng Renilla luciferase (Promega GmbH, Mannheim, Germany) using Lipofectamine 3000 (Thermo Fisher). After 24 h, the cells were treated with NGR1 (50 μM) and BIO (0.5 μM) for 24 h, separately. Subsequently, cells were lysed in 150 μL/well passive phenylbenzothiazole (PPBT) buffer, and the luciferase activity was measured using a Dual-Luciferase^TM^ Reporter Assay System (Promega Corporation, WI, USA). The firefly luciferase activity was normalized to Renilla luciferase activity.

### Wound healing assay

A scratch wound was created using a plastic pipette (10 μL) tip. NCM460 cells were then washed with PBS to remove any debris and treated with either DSS (20 μg/mL) or DSS (20 μg/mL) + NGR1 (100 μM) for 24 h. The width of the scratch was measured using microscopy at 0 and 24 h post-dosing, and the percentage of wound closure was calculated by comparing the scratch width at 24 h to the initial scratch width.

### Flow cytometry

NCM460 cells were treated with either DSS (20 μg/mL) or DSS (20 μg/mL) + NGR1 (100 μM) for 24 h. Then, NCM460 cells were harvested and washed with PBS after experimental treatment. Cells were then suspended in a binding buffer containing Annexin V-fluorescein isothiocyanate (FITC) and propidium iodide (PI), and incubated in the dark at room temperature for 15 min. Flow cytometry analysis was performed to detect apoptotic cells. The data were analyzed using Guava software, and the percentage of apoptotic cells was expressed.

### Statistical analysis

Statistical analysis was performed using GraphPad Prism 9.0 software. Data were presented as mean ± standard deviation (SD). Differences between groups were analyzed using one-way analysis of variance (ANOVA). *P* < 0.05 was considered statistically significant. All experiments were repeated at least three times.

## Results

### NGR1 ameliorated DSS-induced mouse colitis

**Fig. 1:**
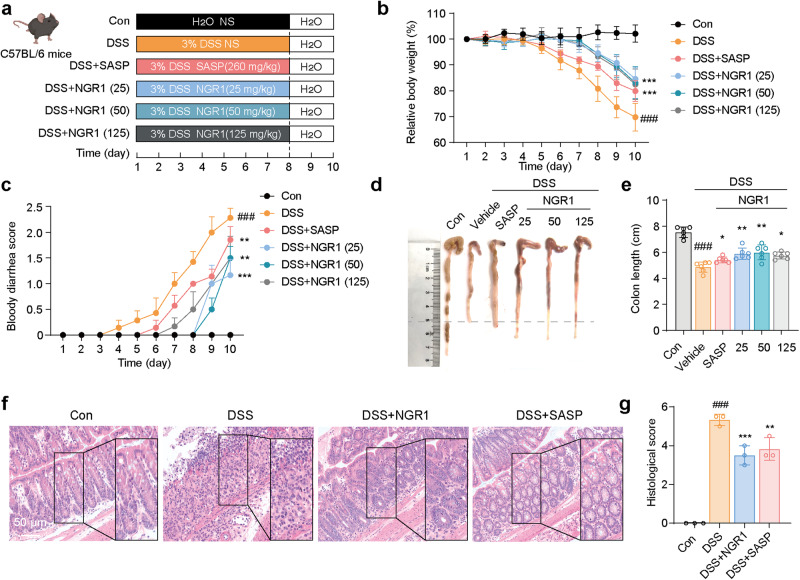
NGR1 relieves the symptoms of colitis mice caused by DSS. **a** The drawing chart presented the animal experimental procedure. **b** Changes in body weight in mice of each group. The data were plotted as a percentage of the weight at baseline. Each bar represents the mean ± SD (*n* = 6 per group). (**c**) The occurrence of bloody diarrhea. The data are plotted as a percentage of the total mice that had bloody diarrhea at different time points. Each bar represents the mean ± SEM (*n* = 6 per group). (**d**, **e**) Colonic-length changes in mice of each group. **f** Colonic pathological injury was examined by H&E staining. Scale bar = 50 μm. **g** Histology scores were counted and presented. Each bar represents the mean ± SD (*n* = 3 per group). ^###^*P* < 0.001 vs. Control group; *P < 0.05, **P < 0.01, ***P < 0.001 vs. DSS group.

Acute colitis model was designed as described previously (Fig. 1a) [26]. Body weight loss is a prominent indicator of colitis severity [11]. As expected, DSS-treated mice exhibited a significant body weight loss compared with the control mice. However, administration of NGR1 (25, 50, or 125 mg/kg) and SASP (positive control drug) remarkably mitigated the weight loss in colitis mice (Fig. 1b). Moreover, DSS led to a significant bloody diarrhea and colon shortening, while oral administration of NGR1 and SASP ameliorated these conditions in colitis mice (Fig. 1c–e). Additionally, DSS resulted in muscle thickening, neutrophil cell infiltration, epithelial structure loss, crypt loss and mucosal ulceration as evidenced by histopathology of colon sections. Mice treated with NGR1 and SASP exhibited less mucosal architecture loss, fewer ulcerations, and less cellular infiltration (Fig. 1f, g). Since all three doses of NGR1 did not exhibit a significant dose-dependent trend, 25 mg/kg treatment group was chosen for the subsequent experiments. Collectively, our data clearly affirm the capacity of NGR1 in mitigating DSS-induced mouse colitis.

### NGR1 alleviated intestinal barrier impairments in colitis mice

**Fig. 2:**
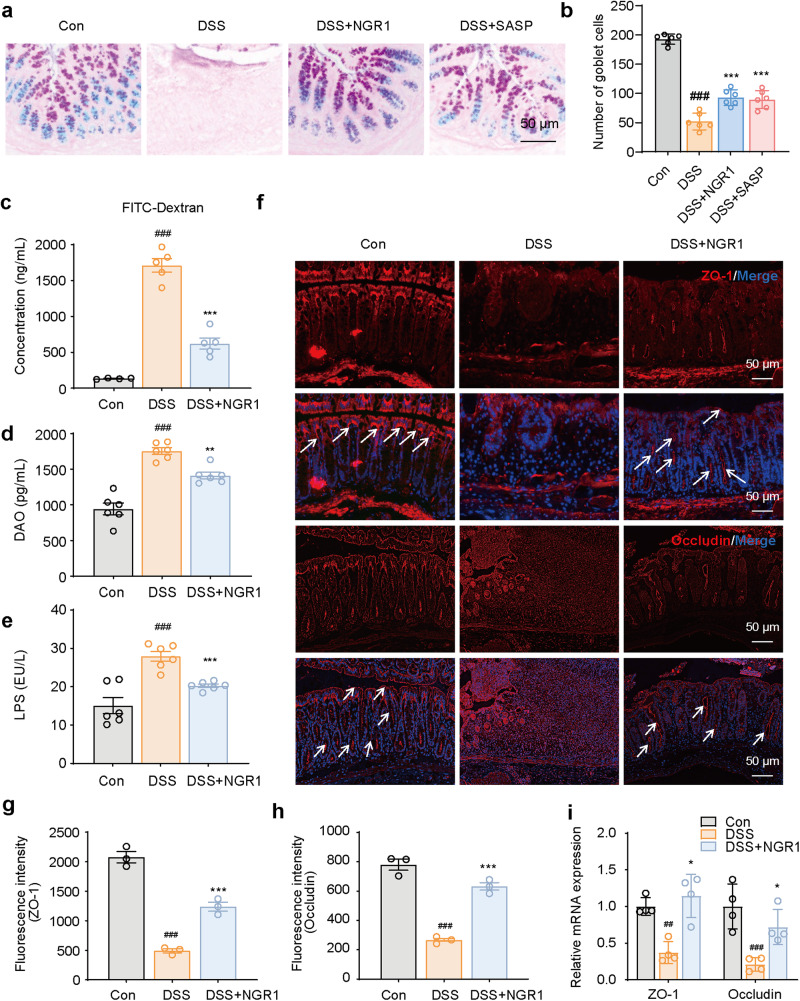
NGR1 enhances intestinal barrier in colitis mice. **a** Colonic pathological injury was examined by AB-PAS staining. Scale bar = 50 μm. **b** Quantitative analysis of goblet cells. **c** The FITC-dextran fluorescence intensity in serum was measured by microplate reader (*n* = 4–5). **d**, **e** DAO and LPS in serum, examined by ELISA. Each bar represents the mean ± SD (*n* = 6 per group). **f** Representative ZO-1 and Occludin immunofluorescence-stained sections of colonic tissue (scale bar = 100 μm). **g**, **h** Relative fluorescence intensity statistics of ZO-1 and Occludin (*n* = 3 per group). **i** mRNA expression levels of ZO-1 and Occludin (*n* = 4 per group). Each bar represents the mean ± SD. ^##^*P* < 0.05, ^###^*P* < 0.001 vs. Control group; *P < 0.05, **P < 0.01, ***P < 0.001 vs. DSS group.

The intestinal barrier, consisting of intestinal epithelial cells, tight junction proteins, and a mucus layer, plays a crucial role in preventing mucosal injury. Impaired barrier function is considered to be strongly linked to colitis [28]. Therefore, the subsequent research focused on elucidating the impact of NGR1 on murine intestinal barrier integrity. Alcian blue (AB) and periodic acid Schiff (PAS) staining of whole colon tissues showed that mucus production and the number of mucus-secreting goblet cells were reduced in mice with DSS-induced colitis compared with healthy mice. However, NGR1 treatment increased the number of the mucus-producing goblet cells and promoted mucus production in colitis mice (Fig. 2a, b). Next, a fluorescein isothiocyanate (FITC)-dextran fluorescence assay was performed to examine intestinal permeability. A high FITC-dextran-positive signal in mice exposed to DSS was observed, indicating that DSS partially destroyed the intestinal barrier; however, the FITC-dextran-positive signal was reduced markedly by NGR1 treatment (Fig. 2c). The same tendency was observed in the serum concentration of lipopolysaccharide (LPS) and diamine oxidase (DAO), both of which are indicators of intestinal barrier injury (Fig. 2d, e). Furthermore, DSS treatment significantly increased mRNA levels and immunostaining-positive signals of tight junction proteins ZO-1 and Occludin in colon, whereas NGR1 treatment prevented all of these changes (Fig. 2f–i). Overall, these data indicated that NGR1 attenuated intestinal barrier injury in DSS-induced colitis mice.

### NGR1 stimulated the proliferation of intestinal crypt cells and facilitated the repair of epithelial injury in colitis mice

**Fig. 3:**
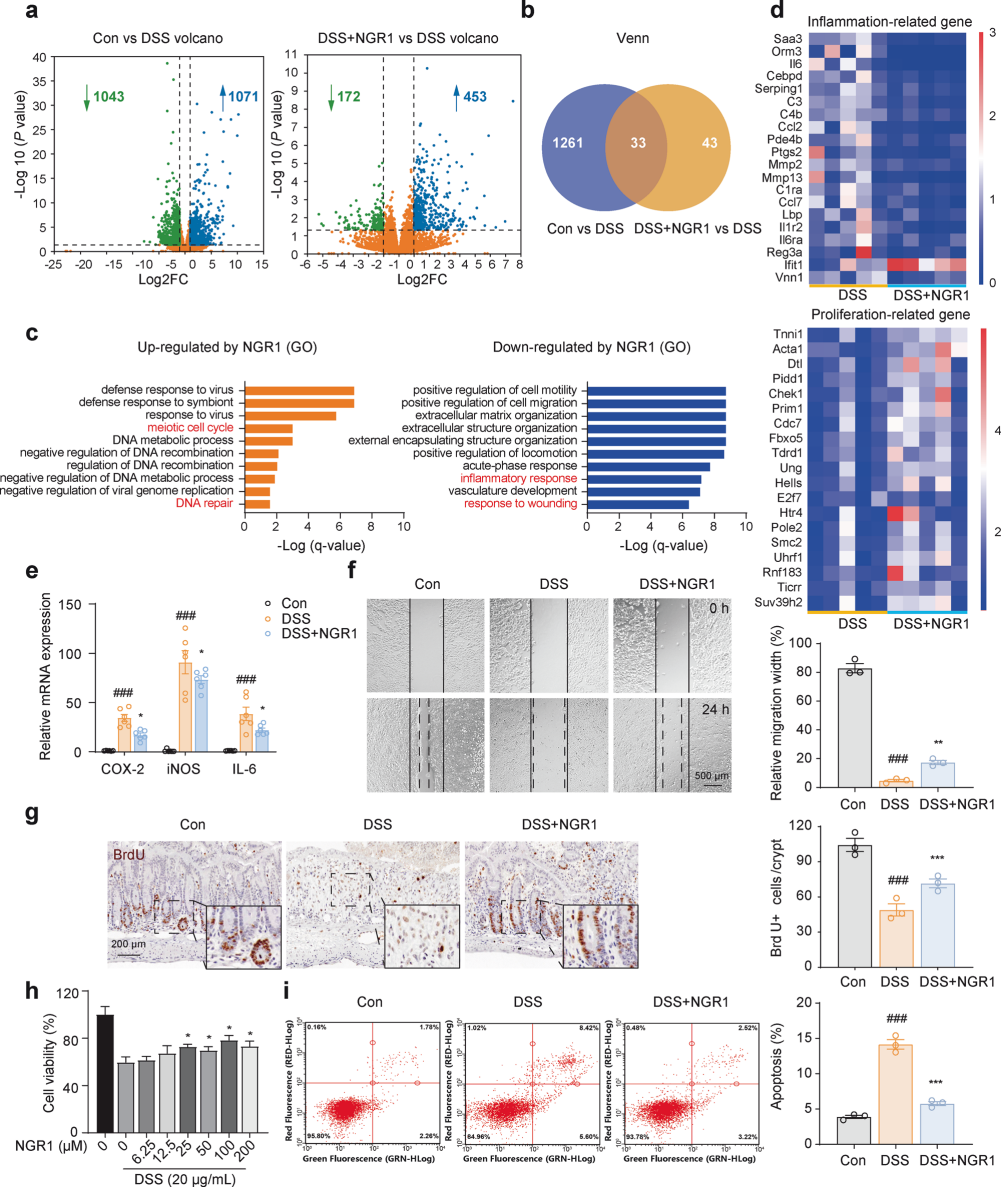
NGR1 remodels the intestinal gene expression profile, downregulates pro-inflammatory genes, and promotes the repair of epithelial injury. **a** Volcano plots of RNA-sequencing of differentially expressed genes in the colon. **b** Venn diagram of the intersection of Control, DSS and DSS + NGR1 group. **c** GO analyses showing up/down-regulated gene clusters after NGR1 treatment. **d** Heatmaps of inflammation and proliferation-related genes (*n* = 5 per group). **e** RT-qPCR analysis of the expression of inflammatory mediators of COX-2, iNOS, and IL-6 in the colon samples of mice. Each bar represents the mean ± SD (*n* = 6 per group). **f** Representative images of scratch wound assays for NCM460 cell monolayers at 0 and 24 h after scratching. Each bar represents the mean ± SD (*n* = 3 per group). **g** Representative BrdU immunohistochemical-stained sections of colonic tissue (scale bar = 200 μm). Each bar represents the mean ± SD (*n* = 3 per group). **h** Effect of NGR1 on the viability of NCM460 cells (*n* = 3 per group). **i** The apoptosis of NCM460 cells was detected by flow cytometry with annexin V-FITC/PI double staining, and the experiment was repeated three times. Each bar represents the mean ± SD (*n* = 3 per group). ^###^*P* < 0.001 vs. Control group; *P < 0.05, **P < 0.01, ***P < 0.001 vs. DSS group.

To investigate the mechanisms underlying NGR1-mediated improvements in intestinal barrier injury, we performed transcriptomic analysis on rectal tissue using RNA-sequencing. Based on significant false-discovery rate (FDR) criteria (fold change >2, *P* < 0.05), the administration of DSS led to a significant downregulation in the expression of 1043 genes and a notable upregulation in the expression of 1071 genes compared to the expression levels observed in the control (vehicle-treated) mice (Fig. 3a). However, DSS + NGR1 treatment significantly downregulated the expression of 172 genes and upregulated 453 genes compared to DSS treatment alone (Fig. 3a). Among 1294 significantly altered genes caused by DSS treatment, 33 genes were recovered by DSS + NGR1 treatment (Fig. 3b). Functional enrichment analyses using Gene Ontology (GO) revealed a reduction in genes associated with the inflammatory response (represented by the blue pillar) in the DSS + NGR1 group compared to the DSS group (Fig. 3c). Heatmap analysis of the data from RNA-sequencing indicated that NGR1 downregulated inflammation-related genes in colonic tissue in colitis mice (Fig. 3d). As expected, RT-qPCR assay demonstrated that NGR1 treatment significantly reduced the mRNA levels of pro-inflammatory mediators COX-2, iNOS, and IL-6 in colitis mice (Fig. 3e).

In addition to suppressing the inflammatory response and downregulating the expression of pro-inflammatory mediators in colitis mice, the Gene Ontology (GO) analysis also revealed an increase in the expression of genes associated with the meiotic cell cycle and DNA repair, as well as a reduction in the expression of genes associated with the response to wounding in the DSS + NGR1 group compared to the DSS group (Fig. 3c). To assess the effects of NGR1 on epithelial injury repair, we conducted a wound healing assay in vitro using NCM460 human colorectal epithelial cells. As expected, DSS treatment significantly inhibited the post-injury repair of NCM460 cells. However, treatment with NGR1 significantly promoted the wound healing of NCM460 cells (Fig. 3f), indicating that NGR1 may enhance the repair of intestinal epithelial injury.

Furthermore, heatmap analysis of the transcriptomic data indicated that NGR1 may upregulate the expression of genes associated with intestinal cell proliferation (Fig. 3d). There is substantial evidence suggesting that intestinal crypt proliferation, a precisely regulated process responsible for epithelial cell turnover, plays a crucial role in repairing the impaired intestinal barrier. When this process is hindered, it can lead to a disruption in intestinal barrier function, subsequently triggering a range of gastrointestinal disorders [15]. To assess the impact of NGR1 on crypt cell proliferation and intestinal epithelial repair, we conducted a BrdU incorporation assay in vivo. The number of BrdU-positive cells was decreased in the colorectal crypt of the DSS group, while NGR1 treatment led to an increased number of proliferating crypt cells (Fig. 3g). This suggests that NGR1 may enhance crypt cell proliferation in DSS colitis mice. Additionally, we evaluated the effects of NGR1 on NCM460 colorectal cell apoptosis using a CCK-8 assay in vitro. NGR1 treatment was found to partially reverse the DSS-induced decline in the cell viability of NCM460 cells (Fig. 3h), providing additional confirmation of NGR1’s pro-proliferative effect on intestinal epithelial cells. Similarly, a cell cytometry assay further confirmed the alleviating effects of NGR1 on DSS-induced apoptosis in NCM460 cells (Fig. 3i).”

Collectively, our results offer valuable insights into the mechanisms of NGR1 against colitis and underscore its potential to ameliorate intestinal barrier dysfunction by inhibiting inflammatory responses, while simultaneously promoting the repair of intestinal epithelial cells through the stimulation of crypt stem cell proliferation.

### NGR1 enhanced the proliferation and differentiation of ISCs in both colitis mice and intestinal organoids

**Fig. 4:**
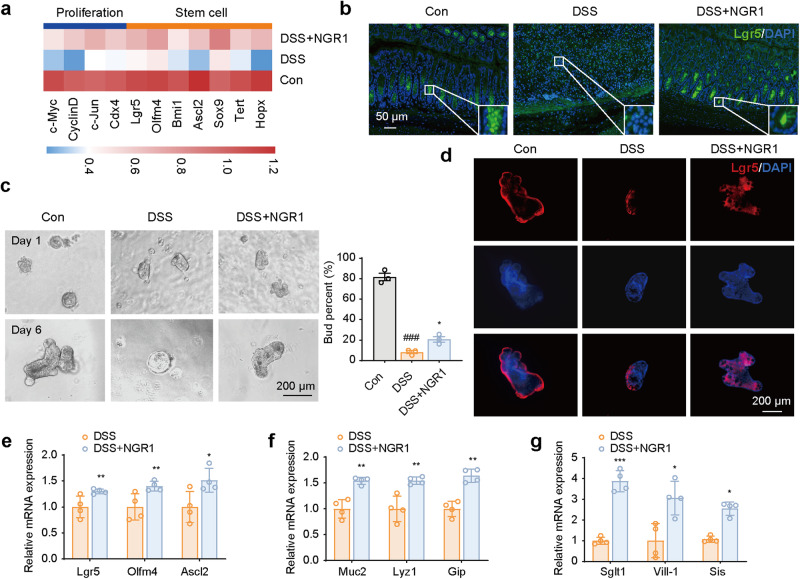
NGR1 protects against the damage of Lgr5^+^ intestinal stem cell induced by DSS. **a** Heatmaps of proliferation associated and stem cell-associated genes (*n* = 6 per group). **b** Representative Lgr5 immunofluorescence-stained sections of colonic tissue (scale bar = 50 μm). **c** Representative budding diagram of intestinal crypt Organoid, and the number of Organoid buds was counted and presented. (scale bar = 200 μm) (*n* = 3 per group). **d** Representative Lgr5 immunofluorescence-stained sections of Organoid. Scale bar = 200 μm. **e**–**g** Effects of NGR1 on the expression of Lgr5, Olfm4, Ascl2, Muc2, Lyz1, Gip, Sglt1, Vill-1, and Sis mRNA in Organoid (*n* = 4 per group). Each bar represents the mean ± SD. ^###^*P* < 0.001 vs. Control group; *P < 0.05, **P < 0.01, ***P < 0.001 *vs*. DSS group.

Given the presence of abundant ISCs within the crypts, where they play a pivotal role in driving intestinal epithelial repair and regeneration through proliferation and differentiation [5], we proceeded to examine the influence of NGR1 on the mRNA expression of ISC markers and relevant proliferation genes in colitis mice. The results revealed that DSS-induced colon injury led to a decrease in mRNA levels of crypt proliferation and stem cell-related genes, including c-Myc, Cyclin D, c-Jun, Cdx4, Lgr5, Olfm4, Bmil1, Ascl2, Sox9, Tert, and Hopx. However, NGR1 treatment effectively restored the decreased expression of these genes (Fig. 4a). Subsequent immunofluorescence staining experiments on colonic tissue confirmed that NGR1 can upregulate the expression of the ISC marker Lgr5 (Fig. 4b).

Intestinal organoids have become a common and valuable tool in IBD research, as they can mimic physiological processes such as the intestinal mucosal barrier and the proliferation and differentiation of crypt stem cells [29]. To further investigate the effects of NGR1 on DSS-induced crypt organoid damage, we constructed a 3D crypt organoid model in vitro. The results revealed that exposure to DSS led to a significant decrease in organoid growth and bud rate when compared to the vehicle-treated group. However, administration of NGR1 effectively mitigated the detrimental effects of DSS on the organoids, resulting in a notable improvement in bud rate (Fig. 4c). Immunofluorescence staining confirmed that NGR1 effectively alleviated the decline in Lgr5 expression in the organoids induced by DSS (Fig. 4d). Furthermore, the expression levels of crypt stem cell differentiation-related genes in the organoids were analyzed. The results suggested that NGR1 treatment upregulated the expression of genes associated with stem cells (Lgr5, Olfm4, Ascl2), secretory cells (Muc2, Lyz1, Gip), and absorptive cells (Sglt1, Vill, Sis) (Fig. 4e–g).

In summary, these results suggest that NGR1 directly promoted the proliferation, growth, and differentiation of ISCs and, as a result, may contribute to the repair of intestinal epithelial injury in DSS colitis mice.

### NGR1 activated the Wnt signaling pathway in both colitis mice and intestinal organoids

**Fig. 5:**
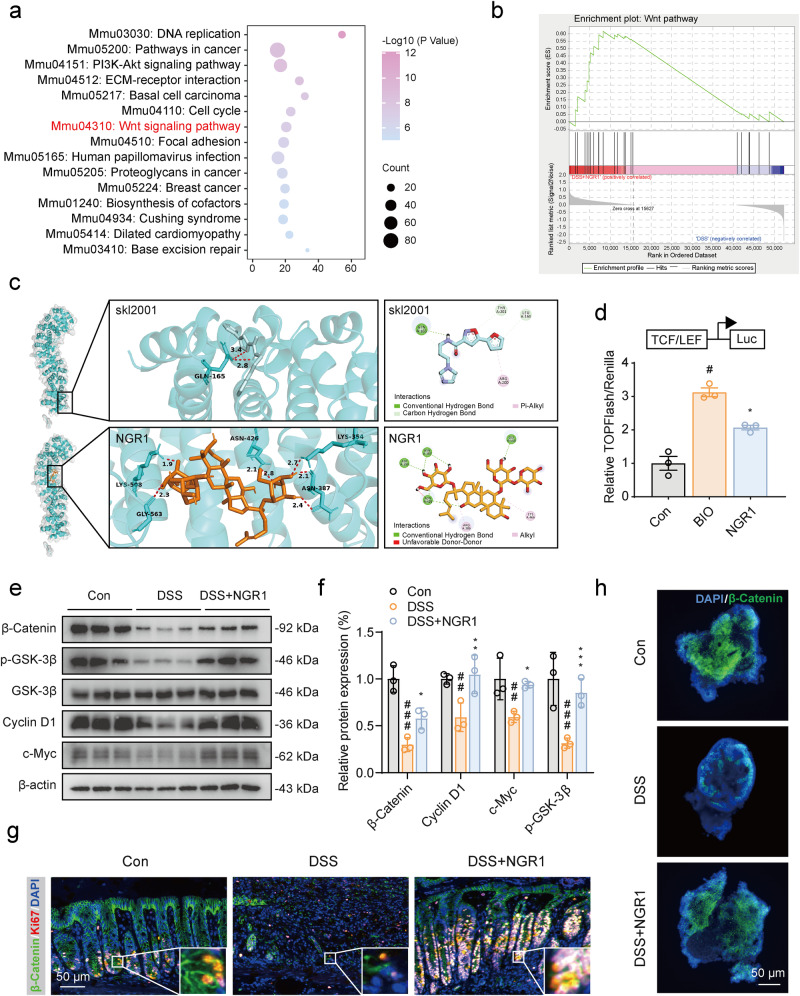
NGR1 activates the Wnt/β-Catenin signaling in colitis mice. **a** The KEGG pathway enrichment analysis of differential genes after NGR1 treatment. **b** GSEA analysis of Wnt signaling pathway-associated genes in DSS + NGR1 group and DSS group mice. Normalized enrichment score (NES) and false discovery rates (FDR) are indicated. **c** Molecular docking of skl2001/NGR1 and β-Catenin proteins and their binding sites. **d** Transcriptional activity of TOPFlash was determined using luciferase reporter assay. **e**, **f** Representative protein expression levels of β-Catenin, p-GSK-3β, Cyclin D1, c-Myc and β-actin. Each bar represents the mean ± SD (*n* = 3 per group). **g** Representative β-Catenin (green) and Ki67 (red) immunofluorescence-stained sections of colonic tissue. **h** Representative β-Catenin immunofluorescence-stained protein expression in Organoid. ^#^*P* < 0.05 ^##^*P* < 0.01, ^###^*P* < 0.001 vs. Control group; *P < 0.05, **P < 0.01, ***P < 0.001 vs. DSS group.

Subsequently, we explored the putative signaling pathway involved in NGR1-mediated ISC proliferation and differentiation. KEGG pathway enrichment analysis indicated significant enrichment of differential genes in pathways related to DNA replication, PI3K-Akt signaling, ECM-receptor interaction, and the Wnt signaling pathway (Fig. 5a). Notably, activation of the Wnt signaling pathway is crucial for maintaining the ISC reservoir and promoting their differentiation into various intestinal cell types, including absorptive enterocytes, goblet cells, and enteroendocrine cells. Dysregulated Wnt pathway modulation has also been implicated in the development of IBD [16]. Therefore, we focused on examining the regulatory impact of NGR1 on the Wnt signaling pathway. We compared the Wnt signaling molecules between DSS group and DSS + NGR1 group using gene set enrichment analysis (GSEA). The results demonstrated a significant upregulation of the Wnt signaling pathway and its core genes in DSS + NGR1 group mice (Fig. 5b).

To delve deeper, we performed molecular docking to evaluate the potential binding of NGR1 to the protein of β-Catenin. As a positive control compound, skl2001 is an agonist of the Wnt/β-Catenin pathway. It combines with β-Catenin to block the interaction between Axin and β-Catenin [13]. This study showed that both NGR1 and skl2001 can bind to the β-Catenin protein with relatively low binding energies of −7.29 and −5.86 kcal/mol, respectively. NGR1 interacted with amino acid residues, including ASP390, ASN387, GLY563, ASN426, ARG386 and CYS466. The agonist (skl2001) interacted with amino acid residues of GLN165, THR201, LEU160 and ARG200 (Fig. 5c). We further assessed the activation of the Wnt signaling pathway using the TOPFlash assay, showing that both the BIO positive control and NGR1 can significantly upregulate the relative activity of TOPFlash/Renilla (Fig. 5d). In addition, Western blot analysis of colonic samples showed that NGR1 increased the expression of several Wnt pathway genes, including β-Catenin, p-GSK-3β, Cyclin D1, and c-Myc (Fig. 5e, f). Immunofluorescence staining confirmed a notable restoration of β-Catenin and the cellular proliferative protein Ki67 in the crypts of colitis mice after NGR1 administration (Fig. 5g). This was further supported by β-Catenin immunofluorescence staining in organoids (Fig. 5h).

These findings demonstrated that NGR1 activated the Wnt signaling pathway in both colitis mice and intestinal organoids, thereby contributing to the regeneration of ISCs following injury induced by DSS.

### NGR1 promoted the regeneration of ISCs in colitis mice through the activation of the Wnt/β-Catenin signaling pathway

**Fig. 6:**
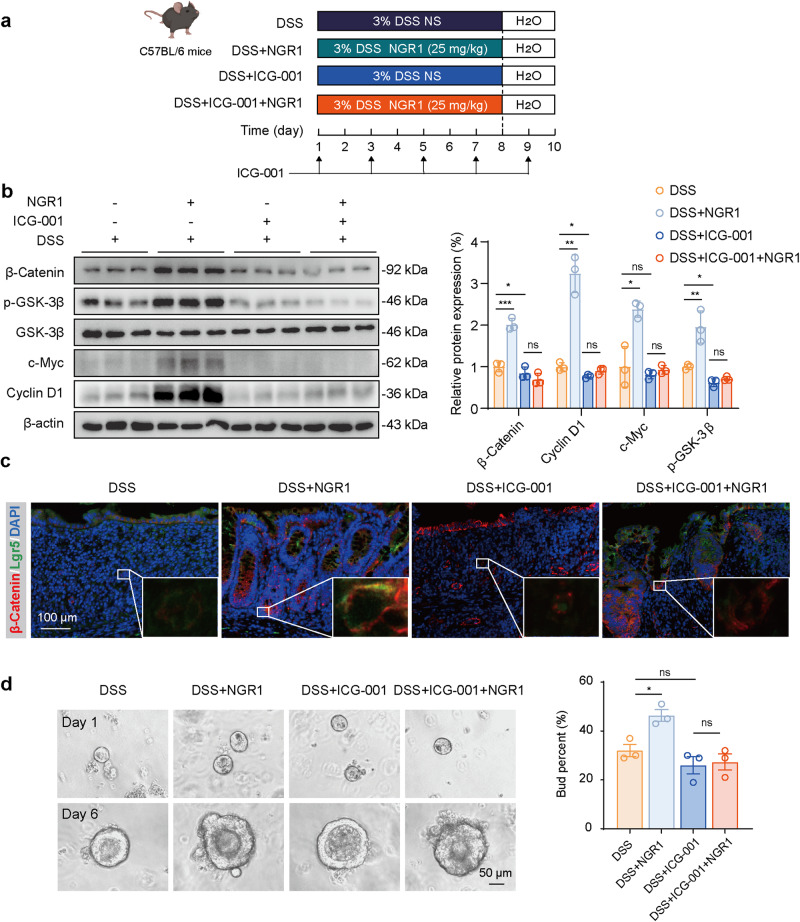
ICG-001 abolishes the protective effect of NGR1 on DSS-induced colitis. **a** The drawing chart presented the animal experimental procedure. **b** Representative protein expression levels of β-Catenin, p-GSK-3β, Cyclin D1, c-Myc and β-actin. Each bar represents the mean ± SD (*n* = 3 per group). **c** Representative Lgr5 (green) and β-Catenin (red) immunofluorescence-stained sections of colonic tissue. **d** Representative budding diagram of intestinal crypt Organoid, and the number of Organoid buds were counted and presented. Each bar represents the mean ± SD (*n* = 3 per group). *P < 0.05, **P < 0.01, ***P < 0.001 vs. DSS group.

**Fig. 7:**
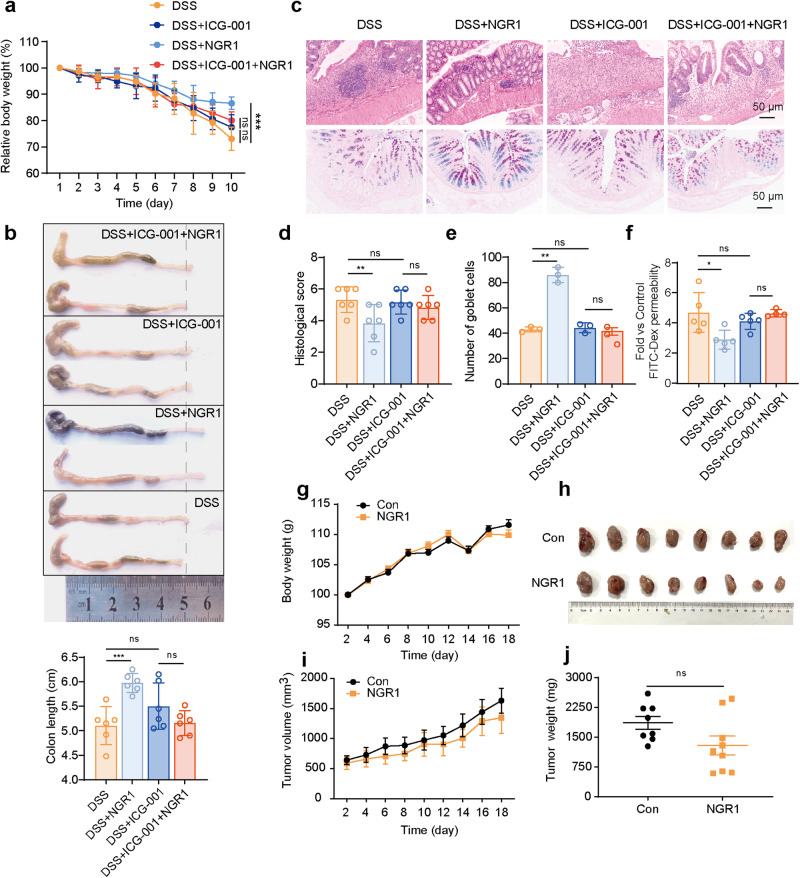
ICG-001 inhibites the activation of Wnt/β-Catenin signaling by NGR1. **a** Changes in body weight in mice of each group. The data were plotted as a percentage of the weight at baseline. Each bar represents the mean ± SD (*n* = 6 per group). **b** Colonic-length changes in mice of each group. Each bar represents the mean ± SD (*n* = 5–6 per group). **c** Colonic pathological injury was examined by H&E staining and AB-PAS staining. Scale bar = 50 μm. **d** Histology scores were counted and presented. **e** Quantitative analysis of goblet cells. **f** The fluorescence intensity of FITC-dextran was detected by fluorescence spectroscopy. Each bar represents the mean ± SD (*n* = 3–6 per group). **g** The mice body weight changes were monitored throughout the study. Each bar represents the mean ± SD (*n* = 8–9 per group). **h** Tumors were taken out after sacrifice of animal. **i** Tumor volume was measured during the test period. Each bar represents the mean ± SD (*n* = 8–9 per group). **j** Tumor weights were measured after the sacrifice of animal. Each bar represents the mean ± SD (*n* = 8–9 per group). *P < 0.05, **P < 0.01, ***P < 0.001 vs. DSS group.

To further investigate the in vivo roles of Wnt/β-Catenin activation, we administered ICG-001, a Wnt/β-Catenin inhibitor that blocks the binding of CBP to β-Catenin, via intraperitoneal injection at a previously established dose [30]. The experiment was designed and conducted following the process outlined in Fig. 6a. ICG-001 treatment was indicated to inhibit the upregulation of β-Catenin, p-GSK-3β, c-Myc, and Cyclin D1 expression induced by NGR1 in colonic tissue (Fig. 6b). The impact of ICG-001 on NGR1’s upregulation of Lgr5 and β-Catenin protein expression was validated through immunofluorescence staining in the crypts (Fig. 6c). NGR1 significantly enhanced colonic crypt Lgr5 and β-Catenin fluorescence, which was effectively abrogated upon the introduction of ICG-001. Furthermore, the introduction of ICG-001 impeded the recovery of organoid budding rate, which had been reduced by DSS, triggered by NGR1 (Fig. 6d). We subsequently assessed whether ICG-001 interferes with the effects of NGR1 on DSS-induced colitis. As shown in Fig. 7a–f, the presence of ICG-001 considerably attenuated the ameliorative effects of NGR1 on colitis mice, as evidenced by reduced improvements in body weight, colon length, histological changes, the number of goblet cells, and the intestinal permeability.

Substantial evidence has suggested that chronic ulcerative colitis is associated with an increased risk of intestinal cancer, which is believed to be linked to excessive activation of the Wnt pathway. Since NGR1 has shown activation of the Wnt signaling pathway, we conducted an investigation to determine whether it promotes cancer development. Using a mouse xenograft model of CT26 colon cancer cells, no evidence was found to support its promotion of colon cancer development (Fig. 7g–j).

Taken together, these findings suggest that NGR1 may, at least partially, mediate the promotion of ISCs regeneration in colitis mice through the upregulation of the Wnt/β-Catenin signaling pathway.

## Discussion

Inflammatory bowel disease (IBD), encompassing both Crohn’s disease and ulcerative colitis, is a complex and debilitating condition characterized by chronic inflammation of the gastrointestinal tract [31]. Recent investigations have shed light on the disease’s underlying mechanisms, revealing aberrant proliferation and differentiation of ISCs, coupled with a profound inflammatory response [8]. Activation of key signaling pathways associated with ISC proliferation and differentiation, such as Wnt, BMP, Hippo, and Notch pathways, has shown promise in colitis models [32]. However, these approaches have yielded disappointing outcomes, primarily due to suboptimal therapeutic efficacy and undesirable treatment-related adverse effects. In this study, we demonstrated that NGR1, a natural phytoestrogen, exerts its beneficial effects on colitis by suppressing inflammation and promoting Lgr5^+^ stem cell and epithelial regeneration through the activation of the Wnt signaling pathway. These findings provide compelling evidence supporting the potential of NGR1 as a promising drug candidate for the treatment of patients with colitis.

In addition to attenuate body weight loss, colon shortening, and histopathological lesions in mouse models of colitis, treatment with NGR1 resulted in a significant improvement in DSS-induced goblet cell injury. This improvement was characterized by an increase in the number of goblet cells in the intestinal lamina propria and enhanced mucus production. Indeed, our previous investigations have revealed that NGR1 has a pronounced ameliorative impact on murine colitis induced by DSS and trinitrobenzene sulfonic acid (TNBS) [25]. This effect is achieved through the activation of the pregnane X receptor (PXR) target, which effectively curtails the excessive activation of NF-κB and mitigates the aberrant intestinal inflammatory response [25]. Interestingly, in this study, we serendipitously discovered the beneficial effects of NGR1 on dysregulated intestinal mucus secretion in colitis mice. This finding suggests that NGR1 may potentially play a regulatory role in the repair of intestinal mucosal barrier damage and epithelial reconstruction.

The intestinal mucosal barrier, a crucial component of gastrointestinal homeostasis, comprises a complex network of interrelated structures and mechanisms [33]. It consists of a single layer of epithelial cells tightly joined by intercellular junctions, including transmembrane proteins (such as Occludin and Claudin) and peripheral proteins (such as ZO protein), which form a physical barrier preventing the uncontrolled passage of luminal contents into the underlying tissues [34]. Additionally, the mucus layer, predominantly composed of mucins secreted by goblet cells, serves as a protective shield against luminal pathogens and toxins [35]. However, when the intestinal mucosal barrier becomes compromised, a phenomenon commonly referred to as “leaky gut,” it results in the translocation of luminal components, including lipopolysaccharides (LPS) and diamine oxidase (DAO), into the bloodstream [36]. Elevated levels of LPS and DAO in the serum have been documented in various studies, providing evidence for the existence of this intestinal permeability in patients with IBD [37]. In this study, we discovered that NGR1 significantly downregulated the elevated levels of serum DAO, LPS, and FITC-Dextran in colitis mice. Additionally, NGR1 administration increased the expression of intestinal ZO-1 and Occludin, key tight junction proteins involved in maintaining epithelial barrier integrity. These findings suggest that treatment with NGR1 may have the potential to modulate the regeneration and functional remodeling of epithelial cells in DSS-induced colitis mice.

To unravel the underlying mechanisms of NGR1, we conducted transcriptome sequencing analysis to disclose the alterations in signaling pathways mediated by NGR1. By analyzing the intestinal gene expression profile regulated by NGR1, we found that NGR1 upregulated DNA repair and meiotic cell cycle in DSS model mice while downregulating the inflammatory response and response to wounding. Subsequently, the heatmap analysis of the relevant genes also confirmed the inhibitory effects of NGR1 on the expression of inflammation-related genes and the significant stimulatory effect on the expression of proliferation-related genes. Moreover, research has shown a downregulation trend of proliferation-related genes, such as c-Myc, Cyclin D, c-Jun, and Ccnd, in patients with IBD [38]. However, NGR1 exhibited an upregulatory effect on the expression of these proliferation-related genes mentioned above.

ISCs are specialized cells residing in the crypts of the intestinal glands and possess the unique ability to self-renew and differentiate into various cell lineages, including absorptive enterocytes, goblet cells, enteroendocrine cells, and Paneth cells [39]. The main function of ISCs is to replenish the epithelial cell population, ensuring the continuous turnover and repair of the intestinal mucosa [40]. Lgr5 has emerged as a well-established marker for actively cycling ISCs located at the base of the intestinal crypts. Lgr5^+^ ISCs are responsible for the continuous regeneration of the intestinal epithelium [41]. Given the regulatory effects of NGR1 on intestinal cell proliferation, it is inferred that there may exist a regulatory and influential effect on ISCs. As speculated, immunofluorescence staining experiments revealed that treatment with NGR1 significantly increased the number of fluorescently labeled cells in the intestinal crypts expressing Lgr5. RT-qPCR analysis further confirmed its upregulatory effect on mRNA expression of key genes associated with ISCs, including Lgr5, Olfm4, Ascl2, Sox9, Tert, and Hopx. However, the exact nature of the upregulatory effect of NGR1 on ISCs, whether it is a direct or indirect effect, remains uncertain.

In this study, we established an intestinal organoid model in vitro to investigate the impact of NGR1 on ISCs. The results demonstrated that NGR1 directly promoted the budding and proliferation of intestinal organoids, as evidenced by increased fluorescence labeling of Lgr5 in the organoids. The enrichment analysis of gene expression profiles in the intestine revealed that NGR1 significantly enriched the Wnt signaling pathway. Activation of the Wnt signaling pathway is considered crucial for maintaining the ISCs pool and promoting their proliferation [42]. Additionally, the Wnt signaling pathway influences cell fate determination, directing the differentiation of ISCs into various lineages, including absorptive enterocytes, goblet cells, and enteroendocrine cells [43, 44]. Furthermore, aberrant regulation of the Wnt pathway has been implicated in intestinal disorders, such as colorectal cancer and IBD [45]. Activation of the Wnt pathway leads to the stabilization and translocation of β-Catenin into the nucleus, where it interacts with transcription factors of the TCF/LEF family to promote the expression of target genes involved in cell cycle progression and self-renewal [46].

The TOPFlash assay experiment further confirmed the direct activating effect of NGR1 on the Wnt signaling pathway. AXIN acts as a central component in the destruction complex, which is responsible for the degradation of β-Catenin [47]. Upon Wnt pathway activation, AXIN is recruited to the Wnt receptor complex, leading to the inhibition of the destruction complex and the stabilization of β-Catenin [47]. Our results revealed that NGR1 bound to specific amino acid residues of the β-Catenin protein. Western blot analysis demonstrated that NGR1 upregulated key proteins, including β-Catenin, p-GSK-3β, Cyclin D1, and c-Myc in vivo, which was further confirmed by immunofluorescence assays. This suggests that the Wnt signaling pathway may mediate the potential of NGR1 to promote ISCs proliferation, differentiation, and epithelial reconstruction. However, a pertinent question arises as to whether NGR1 retains its ameliorative effects on DSS colitis when the Wnt signaling pathway in the intestine is suppressed.

ICG-001, a novel small molecule selective inhibitor of the Wnt/β-Catenin pathway, exhibits an exquisite affinity for the transcriptional coactivator CBP, engaging in a specific and potent binding interaction [48]. Through this extraordinary affinity, ICG-001 disrupts the delicate interplay between CBP and β-Catenin, leading to the suppression of Wnt/β-Catenin-mediated gene transcription [49]. We conducted further investigations to ascertain whether the efficacy of NGR1 in alleviating intestinal inflammation would be compromised or nullified upon the targeted inhibition of the Wnt/β-Catenin pathway by ICG-001. As anticipated, ICG-001, when administered, significantly diminished the beneficial effects of NGR1. This is evidenced by the disappearance of NGR1’s originally observed abilities to ameliorate weight loss, diarrhea, bloody stools, and colon shortening in the DSS mouse model. The impact of ICG-001 on NGR1’s activation of the colonic Wnt signaling pathway was further elucidated through Western blot analysis and immunofluorescence experiments. Moreover, in vitro, intestinal organoid experiments demonstrated that the pro-proliferative effect of NGR1 on ISCs was abolished following the inhibition of the Wnt signaling pathway. These findings underscore the dependence of NGR1’s facilitation of intestinal mucosal and epithelial reconstruction on the Wnt signaling pathway.

A substantial body of research indicates a potential association between the activation of the Wnt signaling pathway and the development of intestinal tumors [50]. Our findings demonstrated that NGR1 exhibited an activating effect on the Wnt signaling pathway, raising concerns about its potential to promote the occurrence and progression of colorectal cancer. However, to our surprise, subcutaneous colorectal cancer xenograft models have shown that NGR1 displays certain inhibitory effects on colorectal cancer. Nevertheless, the precise mechanisms underlying this inhibitory effect await further experimental validation.

In conclusion, our results indicate that NGR1 alleviates DSS-induced colitis in mice by promoting the restoration of Lgr5^+^ stem cells and epithelial integrity, and this effect is mediated, at least partially, through the activation of the Wnt/β-Catenin signaling pathway.

### Supplementary information

Supplementary Information
