# Supplementary figures and images for: Notoginsenoside R1 promotes Lgr5+ stem cell and epithelium renovation in colitis mice via activating Wnt/β-Catenin signaling
Source: Acta Pharmacol Sin. 2024 Mar 15;45(7):1451–65. doi: 10.1038/s41401-024-01250-7 (PMC11192909; doi:10.1038/s41401-024-01250-7)

**Supporting Information**

Theuncropped images of the original Western blot analyses.


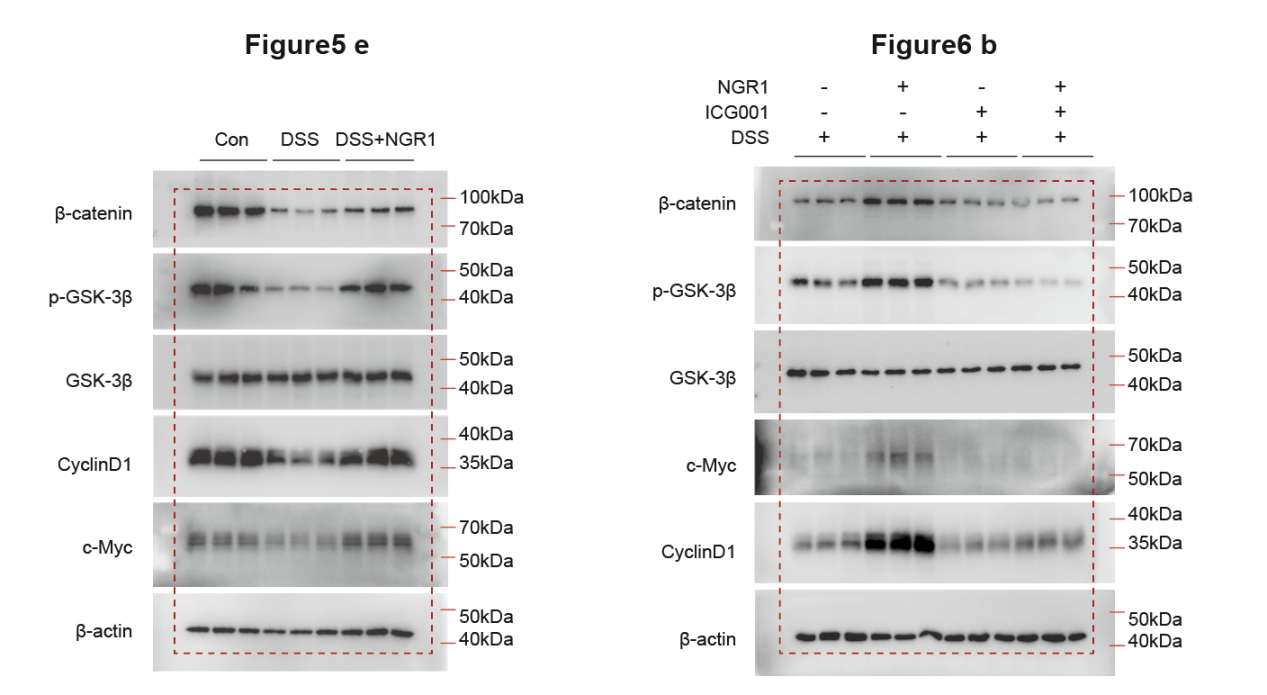

Supplement: Supplementary file 1 — Supplementary Information [file 41401_2024_1250_MOESM1_ESM.docx]
